# Emotional neglect in childhood modulates aperiodic offset in the left pars orbitalis in adulthood

**DOI:** 10.3389/fnins.2025.1719462

**Published:** 2026-01-12

**Authors:** Sota Inoue, Naofumi Otsuru, Hitomi Ikarashi, Koshi Iimuro, Kazuaki Nagasaka, Hiroshi Shirozu, Hideaki Onishi

**Affiliations:** 1 Graduate School, Niigata University of Health and Welfare, Niigata, Japan; 2 Institute for Human Movement and Medical Sciences, Niigata University of Health and Welfare, Niigata, Japan; 3 Department of Physical Therapy, Niigata University of Health and Welfare, Niigata, Japan; 4 Department of Neurosurgery, Functional Neurosurgery Center, Fukuoka Sanno Hospital, Fukuoka, Japan

**Keywords:** abuse, E/I balance, early life stress, magnetoencephalography, neglect

## Abstract

**Introduction:**

Early life stress (ELS) has been identified as a major risk factor for the development of various disorders in adulthood. This study aimed to investigate the impact of ELS on adult brain function by focusing on the aperiodic component (exponent and offset) of neural activity—a novel neurophysiological marker thought to reflect the excitation and inhibition (E/I) balance.

**Methods:**

We recruited 65 healthy adults as participants in this study. Resting-state magnetoencephalography (MEG) data were recorded for 5 min with eyes closed. ELS was assessed using the Childhood Trauma Questionnaire-Japanese version, National Institute of Mental Health (CTQ-JNIMH), and current psychological status was evaluated with the Beck Depression Inventory (BDI) and the State–Trait Anxiety Inventory (STAI). Aperiodic components were extracted from the MEG power spectra using the FOOOF algorithm, and their relationships with CTQ-JNIMH scores were analyzed.

**Results:**

According to the CTQ-JNIMH, 40% of the participants reported experiencing emotional neglect and 27.7% reported physical neglect, while reports of abuse were relatively rare. A significant negative correlation was observed between emotional neglect severity and the aperiodic offset in the left pars orbitalis, which remained significant after false discovery rate correction. In contrast, the aperiodic offset in the left pars orbitalis did not correlate with the current psychological measures (BDI and STAI).

**Discussion:**

These findings suggest that emotional neglect in early life may induce long-lasting alterations in brain function, potentially shifting the E/I balance, as reflected by the aperiodic offset, toward increased excitability in the left pars orbitalis.

## 1 Introduction

Early life stress (ELS) is defined as exposure to various forms of stress during childhood, such as abuse and neglect, and has been associated with an increased risk of developing major psychopathological disorders, including major depression and schizophrenia, in adulthood (Carr et al., 2013; Nemeroff, 2016). In addition to exacerbating the risk for these disorders, ELS has also been associated with poorer treatment responses in depression across various therapeutic approaches (Nanni et al., 2012; Nemeroff, 2016; Nelson et al., 2017). One potential mechanism underlying these adverse effects is long-term alteration in neuroplasticity induced by ELS. Neuroplasticity, which includes processes such as neurogenesis, myelination, synaptogenesis, and synaptic pruning, is particularly active during childhood, playing a crucial role in lifelong brain remodeling (Knudsen, 2004). Since ELS occurs during these critical periods, it can disrupt typical neurodevelopment, leading to persistent structural and functional changes in the brain that persist into adulthood (Teicher and Samson, 2016; Kolb et al., 2017; Malave et al., 2022).

Previous studies have demonstrated that maternal separation stress (MS), a widely utilized animal model of ELS, increases GluR1 and GluR2 expression in the medial prefrontal cortex (mPFC), which may interfere with the process of long-term potentiation (Chocyk et al., 2013). It has also been shown that the MS model induces a reduction in inhibitory neurons in the orbitofrontal cortex and hippocampus (Goodwill et al., 2018; Murthy et al., 2019). Furthermore, MS has been associated with elevated anxiety-like behavior, potentially mediated by downregulation of kainate receptor expression, which is involved in the regulation of GABAergic transmission in the amygdala (Englund et al., 2021). Conversely, a study showed that increased expression of GABA_A α2 receptors in the mPFC and amygdala is associated with enhanced impulsivity in rats with MS (Gondré-Lewis et al., 2016). Collectively, these findings suggest that ELS modulates excitatory and inhibitory functions across various cortical regions.

In humans, brain regions that undergo prolonged postnatal development, such as the prefrontal cortex and hippocampus, have been highlighted as particularly vulnerable to the effects of ELS (Pechtel and Pizzagalli, 2011). A review identified consistent morphological alterations associated with maltreatment, including abuse and/or neglect, in regions such as the anterior cingulate cortex, dorsolateral prefrontal cortex, orbitofrontal cortex, corpus callosum, and hippocampus (Teicher and Samson, 2016). Regarding brain function, previous studies have shown that frontal electroencephalogram (EEG) asymmetry, as indicated by EEG alpha power, is observed in children who have experienced abuse and/or neglect (Miskovic et al., 2009; McLaughlin et al., 2011). Additionally, it has been reported that children raised in environments involving early maternal separation, such as institutional care, exhibited reduced alpha power across the whole brain (Vanderwert et al., 2010). However, no study to date has thoroughly investigated the influence of ELS on adult human brain function from the perspective of excitatory–inhibitory (E/I) balance.

In this context, the aperiodic (1/f-like) component of the power spectral density (PSD) of electrophysiological neural activity has gained increasing attention. The aperiodic component is characterized by two parameters: the offset and exponent. Donoghue et al. showed that the offset is associated with the global power of the aperiodic signal, whereas the exponent reflects the relative distribution of low- and high-frequency activity (Donoghue et al., 2020). In addition, the aperiodic offset is correlated with neuronal population spiking (Manning et al., 2009; Miller et al., 2012) and is increased by the administration of GABA-enhancing drugs, such as diazepam (Gonzalez-Burgos et al., 2023). The aperiodic exponent has been related to the integration of the underlying synaptic currents (Buzsáki et al., 2012), and has been shown to reflect the excitation-inhibition (E/I) balance in the brain (Donoghue et al., 2020). Consequently, the aperiodic component offers a novel perspective for investigating brain function. As mentioned above, animal studies have demonstrated that ELS alters the excitatory–inhibitory (E/I) balance across various brain regions. In the present study, we aimed to elucidate the impact of ELS on adult human brain function by calculating the aperiodic component across the whole brain using magnetoencephalography (MEG).

## 2 Materials and methods

### 2.1 Participants

We recruited 65 right-handed healthy participants (29 males, 46 females, age range, 20–22 years, mean ± standard deviation, 21.1 ± 0.72 years) from the Niigata University of Health and Welfare, who were not taking any medication and had no history of neurological or psychiatric disorders. The required sample size was determined using G*Power (3.1.9.7), based on *a priori* power analysis (two-tailed, Correlation *ρ* H1 = 0.5, *α* err prob. = 0.05, Power (1 − *β* err prob) = 0.95), which indicated a minimum of 46 participants. To account for potential data loss or exclusions, we recruited 65 participants in total. The study was conducted in accordance with the guidelines stipulated in the Declaration of Helsinki and was approved by the ethics committee of Niigata University of Health and Welfare (approval number: 19209–240227).

The exclusion criteria included regular medication use, any neurological or psychiatric disorders, and contraindications to MRI. Furthermore, participants were required to abstain from alcohol for 24 h prior to the MRI and MEG recordings and to avoid caffeine and smoking on the day of the experiment.

### 2.2 Questionnaire for assessing ELS and current mental states

We assessed ELS using the Childhood Trauma Questionnaire-Japanese version, National Institute of Mental Health (CTQ-JNIMH) (Nakajima et al., 2022), developed based on the CTQ-Short Form, a widely utilized questionnaire for assessing ELS (Bernstein and Fink, 1998; Bernstein et al., 2003). The CTQ-JNIMH consists of 28 items that are categorized into five subscales: emotional abuse, physical abuse, sexual abuse, emotional neglect, and physical neglect (each subscale has five items) (Nakajima et al., 2022). Additionally, the questionnaire includes three items designed to detect false negative trauma reports or socially desirable responses. Each item was rated on a 5-point Likert scale ranging from 1 (“never true”) to 5 (“very often true”), with higher scores indicating greater severity of childhood abuse or neglect. The severity classification was based on a previous study by Bernstein and Fink (Bernstein and Fink, 1998). The CTQ-JNIMH scoring criteria are as follows: “Emotional abuse”; None = 5–8, Low = 9–12, Moderate = 13–15, Severe = 16+. “Physical abuse”; None = 5–7, Low = 8–9, Moderate = 10–12, Severe = 13+. “Sexual abuse”; None = 5, Low = 6–7, Moderate = 8–12, Severe = 13+. “Emotional neglect”; None = 5–9, Low = 10–14, Moderate = 15–17, Severe = 18+. “Physical neglect”; None = 5–7, Low = 8–9, Moderate = 10–12, Severe = 13 + .

Furthermore, considering the possibility that ELS may influence current mental status, we assessed depressive symptoms using the Beck Depression Inventory-II (BDI-II) (Beck et al., 1996) and anxiety levels using the State–Trait Anxiety Inventory (STAI) (Spielberger et al., 1971). The BDI-II comprises 21 items, each rated on a 4-point Likert scale, ranging from 0 to 3. The total score ranges from 0 to 63, with higher scores indicating more severe depressive symptoms. The validity and reliability of the Japanese version have also been established (Kojima et al., 2002; Wang and Gorenstein, 2013). The STAI consists of two components: state anxiety, which measures the level of anxiety experienced on the day of the assessment, and trait anxiety, which evaluates an individual’s general tendency toward anxiety as a personality trait. Both components comprise 20 items each, rated on a 4-point Likert scale. Scores for both state and trait anxiety range from 20 to 80, with higher scores indicating greater levels of anxiety. The validity and reliability of the Japanese version of the STAI have been established (Nakazato and Shimonaka, 1989). All questionnaires were administered after the MEG recording.

### 2.3 Magnetoencephalography recording

MEG recordings were conducted in a magnetically shielded room to ensure minimal interference from the external magnetic fields. Magnetic signals were recorded using a helmet-shaped 306-channel detector array (Electa-Neuromag, MEGIN Oy, Helsinki, Finland) with 102 identical triple-sensor elements (102 magnetometers and 204 planar gradiometers). In the present study, we used planar gradiometers. The MEG signals were recorded at a sampling rate of 1,000 Hz, with an online band-pass filter of 0.1–330 Hz applied during acquisition. The participants lay in the supine position during the recording and were instructed to remain still. To calculate the aperiodic component, 5 min of resting-state brain activities were recorded with eye closed.

### 2.4 Magnetic resonance image data acquisition and MEG-MRI co-registration

To estimate the precise sources of the brain activity recorded by MEG, structural MRI scans were acquired from all participants. MRI data acquisition was conducted using a 3.0-T Vantage Galan MRI system (Canon Medical Systems, Japan) equipped with a 32-channel head coil (QD coil, 32ch head SPEEDER, Atlas SPEEDER head/neck). We used a 3D T1-weighted magnetization-prepared rapid gradient echo sequence with the following scan parameters: TE = 2.7 ms, TR = 5.8 ms, voxel size = 0.45 × 0.45 × 0.5 mm, and slice thickness = 1.0 mm. Participants’ heads were immobilized using a head position pad during the scan to ensure image accuracy.

Prior to MEG recording, we placed four or five head position indicator (HPI) coils on the participants’ scalps to detect the head position in the MEG helmet. To align the MEG data with the individual MRI data, we used a three-dimensional (3D) digitizer (Fastrak; Polhemus Navigator Sciences, Colchester, VT, United States) to determine the positions of three anatomical landmarks (the nasion and preauricular points on both sides) as well as the locations of the HPI coils. Additionally, we acquired over 200 points on the head surface using the 3D digitizer to further improve the alignment accuracy. We transformed the digitized point locations into a standardized space to align the MEG data with individual MRI structures. Points located below the nasion were automatically excluded, and an iterative closest point algorithm was applied to optimize the fit between the helmet and the head surface.

### 2.5 MEG data processing

**Figure 1:**
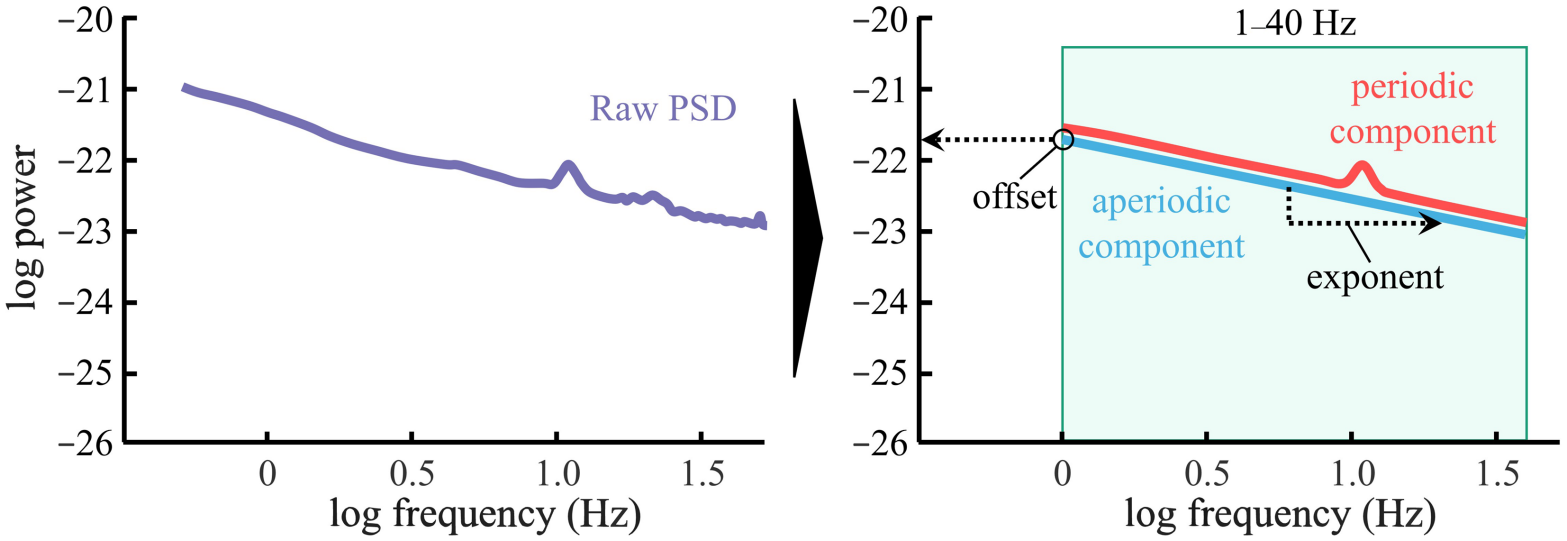
Outline of aperiodic component extraction method. (Left) The PSD before applying the FOOOF model (purple). Transforming both the *x*- and *y*-axes to a logarithmic scale allows the aperiodic component to be approximated by a linear function. (Right) Decomposition of PSD into periodic (red) and aperiodic components (light blue). In the aperiodic component, the intercept represents the offset, and the slope represents the exponent. The shaded light green area indicates the frequency range analyzed in this study, the above values are shown in linear scale.

For the data acquired from the MEG, we employed the signal space separation method using Maxfilter (v.2.2) to eliminate environmental noise (correction limit, 0.98; buffer length, 10 s). The processed data were subsequently imported into Brainstorm on the MATLAB platform for further analysis (Tadel et al., 2011). Initially, we generated PSD estimates from the 204 gradiometers using the Welch method (time window, standardized to 300 s across participants: window length, 2.0 s: window overlap ratio, 50%), and channels with obvious artifacts spanning a wide frequency range were identified as bad channels and excluded from further analysis. Then, we applied the Picard algorithm for Independent Component Analysis (ICA) to create 20 components. From these components, we identified and removed artifacts associated with blinking, eye movements, and electrocardiogram (ECG) noise. Finally, segments containing obvious residual noise, such as electromyographic activity or SQUID jumps, were removed by visual inspection. To estimate the cortical activities, we constructed a head model using the Overlapping Sphere method. Individual T1-weighted structural MRI images were processed using a standard pipeline of a computational anatomy toolbox (CAT12 v12.8.1) within SPM12 (Gaser et al., 2024). For cortical source estimation, the minimum norm estimation (MNE) method was used to calculate the equivalent current dipoles at 15,000 vertices on the cortical surface. We also mitigated MEG-specific noise by incorporating data recorded in an empty room on the same day as participant measurements. Following the source estimation, we applied the Fitting Oscillations & One-Over-F Fitting (FOOOF) model (Frequency range for analysis, 1.0–40 Hz: Peak model, Gaussian: Other setting using default values) (Donoghue et al., 2020). This model allowed for the decomposition of the power spectrum into periodic and aperiodic components, from which the exponent (slope) and offset (intercept) of the aperiodic component were derived (Figure 1). These parameters were computed for the 68 brain regions defined by the Desikan-Killiany atlas, providing regional estimates of the aperiodic exponent and offset values.

### 2.6 Statistical analysis

First, the Shapiro–Wilk test was conducted to assess the normality of all data. As the CTQ-JNIMH subscales for abuse were rarely endorsed by participants, the analyses focused on emotional and physical neglect, which were the subtypes of ELS sufficiently reported in the sample. The Spearman’s correlation was used to investigate the relationship between the aperiodic components (exponent and offset values) in each 68 brain regions and the scores of emotional and physical neglect, respectively. Multiple comparisons were adjusted using the False Discovery Rate (FDR) for the 68 regions defined by the Desikan-Killiany atlas, calculated using the *fdr_bh* function implemented in MATLAB. In addition, Spearman’s correlation was conducted to examine the relationships between emotional and physical neglect scores and current psychological indices, including BDI-II and STAI. These statistical analyses were performed using JASP (v.0.16.4.0). A *p* < 0.05 was considered statistically significant. For nonparametric tests, 95% confidence intervals (CIs) were obtained using 1,000 permutation bootstrap procedures in the JASP.

## 3 Results

### 3.1 Demographic characteristics and descriptive statistics of CTQ-JNIMH, STAI, and BDI-II

The detailed descriptive statistics (mean, standard deviation, and score range), as well as the percentage of participants in each subscale severity for CTQ-JNIMH, are summarized in Table 1. Among the healthy participants in the present study, emotional, physical, and sexual abuse were rarely reported. Specifically, at least “Low” levels of emotional, physical, and sexual abuse were reported by 9.2, 1.5, and 1.5% of participants, respectively. In contrast, emotional and physical neglect were more frequently endorsed, with 40.0 and 27.7% of participants indicating at least “Low” levels of emotional and physical neglect, respectively.

**Table 1:** Descriptive statistics for CTQ-JNIMH and the percentage of participants in each subscale severity category.

CTQ-JNIMH subscale	Mean (± SD) min–max	Percentage of participants in each severity			
		None	Low	Moderate	Severe
Physical Abuse	5.12 (± 0.55) 5–9	98.5%	1.5%	0.0%	0.0%
Emotional Abuse	6.02 (± 1.53) 5–11	90.8%	9.2%	0.0%	0.0%
Sexual Abuse	5.05 (± 0.37) 5–8	98.5%	0.0%	1.5%	0.0%
Physical Neglect	6.49 (± 1.92) 5–12	60.0%	26.2%	10.8%	3.0%
Emotional Neglect	9.14 (± 3.84) 5–20	72.3%	15.4%	12.3%	0.0%

CTQ-JNIMH subscale scores are presented as mean ± standard deviation, followed by the minimum-maximum range.

Considering CTQ-JNIMH distribution and given previous findings that neglect has specific impacts on the development and psychiatric disorders (Jin et al., 2023; Xu et al., 2023), the present study focused subsequent analyses on participants who had experienced physical or emotional neglect.

The BDI and STAI scores, reflecting depressive and anxiety symptoms at the time of MEG recording, are presented in Table 2.

**Table 2:** Descriptive statistics for mental status questionnaires.

Type of questionnaires	Mean (± SD) min–max
BDI-II	3.62 (± 4.69) 0–23
STAI-state	42.63 (± 8.47) 22–58
STAI-trait	42.37 (± 10.31) 24–72

BDI-II and STAI scores are presented as mean ± standard deviation, followed by the minimum-maximum range.

### 3.2 Association between brain aperiodic parameters and CTQ-JNIMH (neglect subscale) scores

**Figure 2:**
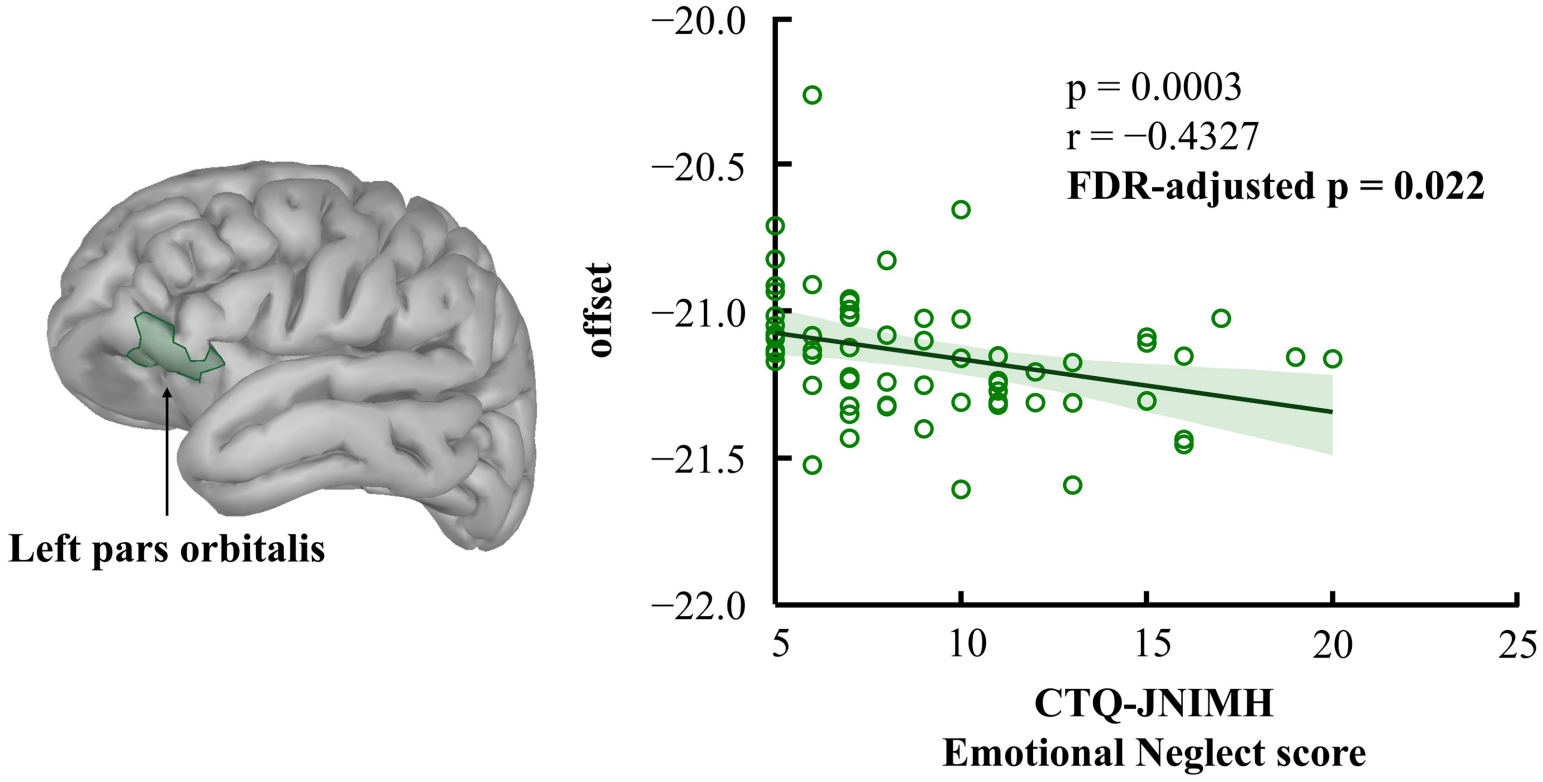
Correlation between offset values in the left pars orbitalis and CTQ-JNIMH emotional neglect score. Spearman’s correlation coefficient was used to examine the relationship between the CTQ-JNIMH emotional neglect score and the aperiodic offset value. The results indicated that participants with higher emotional neglect scores tended to have lower offset values in the left pars orbitalis. This relationship remained significant after FDR correction (FDR-adjusted *p* < 0.05). The shaded area in the figure represents the 95% confidence interval, which was estimated using 1,000 bootstrap resamples.

Spearman’s correlation coefficient was used to examine the association between individuals’ brain aperiodic parameters (exponent and offset) and emotional and physical neglect scores assessed by CTQ-JNIMH, respectively (Table 3). As shown in Figure 2, the offset value in the left pars orbitalis was significantly correlated with the CTQ-JNIMH emotional neglect score [*r* = −0.43, *p* < 0.001, Bootstrapped 95% CI (−0.23, −0.60)], showing a negative correlation. This relationship remained significant after FDR correction (FDR-adjusted *p* = 0.022). In contrast, no significant relationships were found between emotional neglect and the exponent in any of the brain regions. Furthermore, neither the exponent nor the offset showed significant relationships with physical neglect in any brain region.

**Table 3:** Descriptive statistics of aperiodic parameters for all 68 Desikan-Killian cortical regions and their associations with physical and emotional neglect.

Desikan-Killiany atlas brain regions	Offset mean (± SD) min–max	Exponent mean (± SD) min–max	Correlation with physical neglect		Correlation with emotional neglect	
			Offset rho-value uncorrected *p*-value	Exponent rho-value uncorrected *p*-value	Offset rho-value uncorrected *p*-value	Exponent rho-value uncorrected *p*-value
Bankssts L	−21.35 (± 0.24) −21.81 to −20.43	0.97 (± 0.14) 0.72–1.51	0.05 0.69	0.17 0.17	−0.20 0.11	−0.01 0.93
Bankssts R	−21.38 (± 0.24) −21.78 to −20.19	0.97 (± 0.16) 0.68–1.75	0.08 0.51	0.06 0.61	−0.06 0.62	−0.12 0.34
Caudalanteriorcingulate L	−21.87 (± 0.23) −22.32 to −21.11	0.92 (± 0.13) 0.61–1.31	0.10 0.44	0.17 0.17	−0.07 0.59	−0.14 0.26
Caudalanteriorcingulate R	−21.91 (± 0.24) −22.34 to −21.02	0.92 (± 0.13) 0.64–1.38	0.07 0.58	0.11 0.38	−0.03 0.83	−0.16 0.22
Caudalmiddlefrontal L	−21.50 (± 0.19) −21.86 to −20.92	0.88 (± 0.11) 0.61–1.22	0.08 0.52	0.19 0.13	−0.11 0.40	−0.05 0.69
Caudalmiddlefrontal R	−21.52 (± 0.19) −21.92 to −20.83	0.87 (± 0.12) 0.60–1.32	0.13 0.32	0.23 0.06	0.07 0.61	−0.01 0.91
Cuneus L	−21.56 (± 0.26) −22.05 to −20.55	0.89 (± 0.14) 0.69–1.52	0.06 0.62	0.13 0.30	−0.14 0.26	−0.14 0.26
Cuneus R	−21.53 (± 0.26) −22.00 to −20.67	0.89 (± 0.14) 0.66–1.48	0.15 0.23	0.14 0.28	−0.08 0.54	−0.18 0.14
Entorhinal L	−20.99 (± 0.29) −21.53 to −19.76	0.95 (± 0.16) 0.69–1.59	0.03 0.79	0.13 0.30	−0.17 0.19	−0.13 0.31
Entorhinal R	−21.01 (± 0.30) −21.48 to −19.42	0.96 (± 0.17) 0.47–1.83	−0.04 0.72	0.01 0.95	−0.16 0.20	−0.18 0.14
Frontalpole L	−21.27 (± 0.28) −21.81 to −20.15	0.87 (± 0.13) 0.65–1.41	−0.23 0.06	0.19 0.12	−0.37 2.15 × 10^−3^	−0.08 0.52
Frontalpole R	−21.28 (± 0.24) −21.70 to −20.44	0.88 (± 0.13) 0.63–0.34	−0.02 0.87	0.12 0.09	−0.30 0.01	−0.14 0.25
Fusiform L	−21.09 (± 0.25) −21.59 to −20.11	0.92 (± 0.15) 0.66–1.50	0.05 0.68	0.05 0.67	−0.14 0.28	−0.12 0.36
Fusiform R	−21.07 (± 0.30) −21.64 to −19.55	0.95 (± 0.17) 0.60–1.83	−0.10 0.45	−0.06 0.66	−0.25 0.04	−0.28 0.02
Inferiorparietal L	−21.41 (± 0.20) −21.73 to −20.84	0.88 (± 0.12) 0.66–1.21	0.10 0.43	0.15 0.22	−0.11 0.38	0.00 1.00
Inferiorparietal R	−21.42 (± 0.22) −21.76 to −20.48	0.88 (± 0.13) 0.66–1.50	0.13 0.29	0.13 0.31	0.04 0.76	−0.12 0.35
Inferiortemporal L	−20.99 (± 0.24) −21.45 to −20.15	0.93 (± 0.13) 0.70–1.42	0.03 0.79	0.06 0.66	−0.15 0.23	−0.16 0.20
Inferiortemporal R	−21.95 (± 0.28) −21.32 to −19.29	0.95 (± 0.17) 0.74–1.92	−0.06 0.64	−0.04 0.75	−0.25 0.05	−0.26 0.04
Insula L	−21.64 (± 0.24) −22.12 to −20.75	0.97 (± 0.14) 0.72–1.40	0.05 0.72	0.17 0.17	−0.18 0.16	−0.15 0.24
Insula R	−21.63 (± 0.24) −22.06 to −20.47	0.99 (± 0.16) 0.69–1.77	1.99 × 10^−3^ 0.99	0.12 0.35	−0.18 0.14	−0.20 0.12
Isthmuscingulate L	−21.55 (± 0.31) −22.14 to −20.07	0.90 (± 0.17) 0.62–1.74	−0.03 0.83	0.09 0.49	−0.09 0.47	−0.19 0.13
Isthmuscingulate R	−21.51 (± 0.33) −22.26 to −19.93	0.91 (± 0.17) 0.66–1.84	5.09 × 10^−4^ 1.00	0.05 0.69	−0.09 0.48	−0.23 0.07
Lateraloccipital L	−21.17 (± 0.25) −21.68 to −20.35	0.84 (± 0.14) 0.61–1.36	−0.01 0.93	0.01 0.96	−0.21 0.09	−0.21 0.09
Lateraloccipital R	−21.16 (± 0.25) −21.72 to −20.34	0.86 (± 0.14) 0.64–1.40	5.55 × 10^−3^ 0.96	0.03 0.76	−0.12 0.32	−0.20 0.12
Lateralorbitofrontal L	−21.26 (± 0.22) −21.71 to −20.34	0.94 (±)0.14 0.66–1.44	−7.43 × 10^−3^ 0.95	0.17 0.17	−0.33 6.44 × 10^−3^	−0.15 0.23
Lateralorbitofrontal R	−21.25 (± 0.23) −21.64 to −20.13	0.95 (± 0.16) 0.68–1.71	−0.02 0.87	0.14 0.27	−0.30 0.01	−0.21 0.10
Lingual L	−21.35 (± 0.31) −21.95 to −19.86	0.90 (± 0.17) 0.69–1.73	−0.05 0.68	0.03 0.81	−0.15 0.24	−0.15 0.22
Lingual R	−21.35 (± 0.31) −21.93 to −19.93	0.92 (± 0.16) 0.70–1.76	−9.58 × 10^−3^ 0.94	0.10 0.44	−0.14 0.24	−0.18 0.15
Medialorbitofrontal L	−21.20 (± 0.23) −21.63 to −20.06	0.93 (± 0.15) 0.68–1.59	−0.07 0.58	0.13 0.29	−0.23 0.07	−0.17 0.18
Medialorbitofrontal R	−21.22 (± 0.24) −21.54 to −20.02	0.93 (± 0.16) 0.68–1.67	−0.04 0.77	0.17 0.18	−0.24 0.06	−0.16 0.19
Middletemporal L	−21.09 (± 0.21) −21.46 to −20.40	0.94 (± 0.12) 0.71–1.37	0.11 0.37	0.17 0.17	−0.21 0.09	−0.10 0.43
Middletemporal R	−21.08 (± 0.21) −21.40 to −20.00	0.95 (± 0.14) 0.72–1.71	−4.79 × 10^−3^ 0.97	−0.01 0.96	−0.19 0.13	−0.22 0.07
Paracentral L	−21.71 (± 0.24) −22.11 to −20.93	0.88 (± 0.12) 0.48–1.35	0.19 0.13	0.18 0.14	0.09 0.48	−0.08 0.53
Paracentral R	−21.71 (± 0.25) −22.21 to −20.53	0.89 (± 0.14) 0.62–1.66	0.13 0.30	0.18 0.15	0.03 0.79	−0.08 0.50
Parahippocampal L	−21.16 (± 0.27) −21.70 to −19.95	0.90 (± 0.15) 0.59–1.51	0.01 0.93	0.04 0.73	−0.16 0.19	−0.13 0.32
Parahippocampal R	−21.16 (± 0.30) −21.71 to −19.77	0.93 (± 0.16) 0.51–1.71	0.03 0.83	0.05 0.68	−0.09 0.45	−0.12 0.33
Parasopercularis L	−21.40 (± 0.18) −21.83 to −20.89	0.90 (± 0.11) 0.60–1.23	−3.93 × 10^−3^ 0.98	0.16 0.20	−0.23 0.06	−0.09 0.46
Parasopercularis R	−21.45 (± 0.18) −21.71 to −20.80	0.93 (± 0.13) 0.55–1.36	0.13 0.30	0.17 0.16	−0.12 0.34	−0.14 0.28
Parasorbitalis L	−21.15 (± 0.22) −21.61 to −20.27	0.91 (± 0.13) 0.68–1.31	−0.10 0.42	0.09 0.46	**−0.43** **3.18 × 10**^**−4**^ *	−0.23 0.07
Parsorbitalis R	−21.15 (± 0.22) −21.53 to −20.14	0.92 (± 0.15) 0.57–1.63	−0.05 0.67	0.05 0.69	−0.27 0.03	−0.22 0.08
Parstriangularis L	−21.32 (± 0.19) −21.79 to −20.57	0.90 (± 0.12) 0.64–1.27	−0.05 0.68	0.24 0.05	−0.32 9.41 × 10^−3^	−0.10 0.44
Parstriangularis R	−21.35 (± 0.18) −21.63 to −20.67	0.91 (± 0.12) 0.56–1.40	0.02 0.86	0.20 0.12	−0.19 0.12	−0.16 0.20
Pericalcarine L	−21.55 (± 0.29) −22.10 to −20.33	0.91 (± 0.15) 0.67–1.58	0.07 0.60	0.10 0.42	−0.10 0.44	−0.17 0.18
Pericalcarine R	−21.50 (± 0.27) −22.01 to −20.61	0.91 (± 0.14) 0.70–1.46	−0.01 0.93	0.12 0.35	−0.14 0.27	−0.17 0.17
Postcentral L	−21.42 (± 0.18) −21.81 to −20.82	0.87 (± 0.12) 0.52–1.23	0.10 0.42	0.23 0.06	−0.11 0.38	−0.05 0.69
Postcentral R	−21.45 (± 0.21) −21.80 to −20.51	0.88 (± 0.13) 0.52–1.53	0.13 0.32	0.12 0.33	0.05 0.70	−0.15 0.23
Posteriorcingulate L	−21.85 (± 0.30) −22.42 to −20.75	0.91 (± 0.15) 0.67–1.55	0.13 0.31	0.18 0.15	−0.04 0.74	−0.17 0.18
Posteriorcingulate R	−21.85 (± 0.31) −22.42 to −20.62	0.91 (± 0.15) 0.63–1.61	0.02 0.88	0.10 0.43	−0.11 0.37	−0.20 0.12
Precentral L	−21.48 (± 0.19) −21.83 to −20.75	0.89 (± 0.11) 0.66–1.28	0.06 0.62	0.19 0.12	−0.14 0.26	−0.07 0.57
Precentral R	−21.51 (± 0.20) −21.88 to −20.59	0.89 (± 0.12) 0.62–1.52	0.14 0.27	0.15 0.22	0.03 0.79	−0.13 0.31
Precuneus L	−21.66 (± 0.28) −22.19 to −20.54	0.91 (± 0.14) 0.68–1.60	0.12 0.32	0.11 0.40	−0.03 0.84	−0.19 0.12
Precuneus R	−21.66 (± 0.28) −22.13 to −20.47	0.92 (± 0.15) 0.66–1.68	0.08 0.51	0.12 0.36	−0.03 0.79	−0.18 0.15
Rostralanteriorcingulate L	−21.54 (± 0.25) −22.06 to −20.52	0.96 (± 0.16) 0.68–1.58	−0.02 0.90	0.18 0.16	−0.31 0.01	−0.19 0.14
Rostralanteriorcingulate R	−21.54 (± 0.25) −21.89 to −20.35	0.96 (± 0.16) 0.71–1.73	0.05 0.72	0.16 0.20	−0.21 0.09	−0.21 0.10
Rostralmiddlefrontal L	−21.37 (± 0.20) −21.82 to −20.54	0.87 (± 0.14) 0.54–1.29	−0.02 0.84	0.23 0.06	−0.35 3.86 × 10^−3^	−0.06 0.63
Rostralmiddlefrontal R	−21.39 (± 0.18) −21.70 to −20.58	0.87 (± 0.12) 0.54–1.31	0.05 0.71	0.20 0.11	−0.17 0.17	−0.09 0.45
Superiorfrontal L	−21.56 (± 0.18) −21.95 to −20.85	0.87 (± 0.11) 0.61–1.28	0.10 0.44	0.23 0.07	−0.09 0.46	−0.04 0.74
Superiorfrontal R	−21.57 (± 0.19) −21.98 to −20.67	0.86 (± 0.12) 0.51–1.40	0.14 0.26	0.22 0.08	−2.00 × 10^−3^ 0.98	−0.07 0.58
Superiorparietal L	−21.51 (± 0.22) −21.86 to −20.76	0.85 (± 0.11) 0.62–1.36	0.11 0.37	0.20 0.11	−0.06 0.66	−0.06 0.65
Superiorparietal R	−21.52 (± 0.24) −21.95 to −20.68	0.86 (± 0.12) 0.63–1.43	0.13 0.30	0.14 0.27	0.07 0.60	−0.15 0.22
Superiortemporal L	−21.26 (± 0.19) −21.63 to −20.56	0.95 (± 0.13) 0.72–1.40	0.07 0.60	0.20 0.11	−0.21 0.09	−0.11 0.40
Superiortemporal R	−21.24 (± 0.22) −21.54 to −20.03	0.97 (± 0.15) 0.67–1.80	−0.02 0.86	0.09 0.50	−0.20 0.11	−0.19 0.13
Supramarginal L	−21.42 (± 0.19) −21.84 to −20.84	0.90 (± 0.12) 0.59–1.23	0.12 0.33	0.22 0.07	−0.12 0.34	−0.02 0.87
Supramarginal R	−21.47 (± 0.19) −21.83 to −20.68	0.90 (± 0.13) 0.55–1.47	0.14 0.26	0.13 0.30	0.08 0.51	−0.08 0.51
Temporalpole L	−20.96 (± 0.27) −21.49 to −19.88	0.95 (± 0.15) 0.68–1.50	0.08 0.54	0.13 0.28	−0.12 0.34	−0.16 0.21
Temporalpole R	−20.94 (± 0.31) −21.43 to −19.16	0.96 (± 0.19) 0.64–2.00	−0.09 0.47	−0.07 0.60	−0.29 0.02	−0.25 0.05
Transversetemporal L	−21.50 (± 0.22) −21.91 to −20.79	1.00 (± 0.14) 0.71–1.42	0.07 0.60	0.17 0.19	−0.20 0.11	−0.09 0.50
Transversetemporal R	−21.49 (± 0.26) −21.88 to −20.19	1.03 (± 0.17) 0.73–1.87	2.08 × 10^−3^ 0.99	0.17 0.18	−0.07 0.58	−0.08 0.52

Aperiodic parameters (offset and exponent) are presented as mean ± standard deviation, followed by the minimum-maximum range. Cortical regions are defined according to the 68-region Desikan-Killiany atlas. Correlations with Physical and Emotional neglect scores were assessed using Spearman’s correlation. *P*-values were corrected for multiple comparisons using the Benjamini-Hochberg procedure implemented in MATLAB (fdr_bh). Significant areas (FDR-adjusted *p* < 0.05) are shown in bold and with an asterisk.

### 3.3 The relationship between the left pars orbitalis aperiodic component, the psychological questionnaire, and childhood neglect

Given the observed association between emotional neglect and the aperiodic component in the left pars orbitalis, this region was selected for further analysis to explore its relevance to current psychological states. Spearman’s correlation analysis was conducted between the aperiodic offset and exponent in this region and scores on the STAI and BDI-II, but no significant associations were observed (all *p*s > 0.05). Similarly, emotional and physical neglect scores showed no significant correlations with any of the psychological measures (all *p*s > 0.05).

## 4 Discussion

In the present study, approximately 40% of the participants reported emotional neglect. Neglect is recognized as the most frequently experienced form of ELS (Sheikhattari et al., 2006; Gilbert et al., 2009; Salokangas, 2022). The prevalence of neglect observed in our participants aligns with rates reported in previous studies involving college students (Fu et al., 2018). In this study, we focused on the experiences of childhood neglect and investigated their impacts on cortical activity by analyzing the aperiodic component, which has been proposed as a marker of the E/I balance. Our results demonstrated a significant negative correlation between aperiodic offset values in the left pars orbitalis and self-reported emotional neglect. These results suggest that childhood experiences of emotional neglect may lead to functional alterations in specific areas of the prefrontal cortex. While the physiological meaning of the offset value (the intercept of the aperiodic component) is less clearly defined than that of the exponent, it is commonly thought to index neuronal population firing (Manning et al., 2009). However, previous studies have demonstrated an association between the aperiodic offset and the function of glutamate and GABA, both of which are involved in the regulation of E/I balance. In animal studies, the aperiodic offset has been shown to increase following administration of diazepam, a drug that enhances GABAergic activity (Gonzalez-Burgos et al., 2023; Salvatore et al., 2024). Moreover, a human study using magnetic resonance spectroscopy (MRS) has reported that higher aperiodic offset values are associated with lower glutamate concentrations (Glica et al., 2025). These observations imply that a lower aperiodic offset may be indicative of reduced GABAergic influence and/or relatively increased glutamatergic activity. Taken together, these findings indicate that greater childhood emotional neglect is associated with an altered neurotransmitter balance in the left pars orbitalis, potentially reflecting reduced GABAergic function or increased glutamatergic activity. Nevertheless, it is important to note that some studies have suggested an association between higher aperiodic offset values and increased excitatory activity (Winawer et al., 2013; Arutiunian et al., 2024).

A growing body of evidence supports the notion that ELS modulates inhibitory function. A previous study using the MS model, a mouse model of ELS, demonstrated a reduction in parvalbumin (PV)-positive neurons, which are inhibitory interneurons, in the orbitofrontal cortex (Goodwill et al., 2018). This reduction in PV-positive neurons due to ELS has also been demonstrated in other studies (Holland et al., 2014; Irie et al., 2023). Additionally, a human study demonstrated that early institutionalization—an environment characterized by caregiver deprivation and mother–child separation—was associated with reduced alpha power, including the frontal region, which is thought to reflect inhibitory processes (Vanderwert et al., 2010). Consistent with this, fMRI studies also indicate that ELS modulates neural activity related to behavioral inhibition in the frontal cortex (Mueller et al., 2010). Interestingly, individuals with depression or schizophrenia, both of which are frequently observed among those with a history of ELS (Carr et al., 2013; Nemeroff, 2016), have been reported to show lower offset values (Earl et al., 2024; Woronko et al., 2025). Notably, in patients with depression, lower offset values have been found to be associated with the number of depressive episodes, suggesting that chronicity may contribute to further reductions in offset (Woronko et al., 2025). Although further research is needed to deepen our understanding of the aperiodic offset, the present findings highlight the potential impact of ELS on neural mechanisms and support the relevance of offset values as a neurophysiological marker.

Furthermore, the left pars orbitalis, where we observed a reduction in aperiodic offset, possesses compelling functional relevance to the impact of ELS. Prefrontal cortical regions, including the orbitofrontal cortex, are particularly vulnerable to ELS, frequently exhibiting both structural and functional alterations (Davey et al., 2008; Pechtel and Pizzagalli, 2011; Shaw et al., 2020). Previous studies have suggested that the left pars orbitalis contributes to functions such as emotion regulation and response inhibition (Dillo et al., 2010; Belyk et al., 2017). Consistent with this, patients with damage to the left pars orbitalis have been reported to show impaired performance on the Go/NoGo task, a commonly used measure of inhibitory control (Swick et al., 2008). Given its functional role, the observed emotional neglect-related reduction in aperiodic offset in the left pars orbitalis may reflect compromised inhibitory capacity and impaired emotion regulation. Supporting this interpretation, individuals with a history of ELS have demonstrated diminished performance on response inhibition tasks (Mueller et al., 2010; Wu et al., 2021). Such impairments in inhibitory control are also frequently observed in ELS-related psychiatric disorders, including depression, ADHD, and schizophrenia (Weisbrod et al., 2000; Kaiser et al., 2003; Inoue et al., 2012). Collectively, these findings suggest that functional disruption of the left pars orbitalis may underlie the deficits in cognitive control and emotion regulation commonly observed in individuals exposed to early adverse experiences. However, the reduced aperiodic offset in the left pars orbitalis observed in the present study was not directly associated with psychological scales such as the BDI or STAI. Therefore, this finding does not necessarily reflect a negative impact of ELS, but could instead represent a positive adaptation.

A limitation of the present study is its cross-sectional design. Consequently, brain function was only assessed at a single time point, and changes over time remain unknown. Brain function is known to change in response to experience and environmental factors. Recent studies have demonstrated that the aperiodic component (exponent, offset) undergoes changes with aging (Hill et al., 2022; McSweeney et al., 2023). Therefore, to gain a more comprehensive understanding of how ELS affects brain function across the lifespan, future studies should examine developmental trajectories from childhood through old age. A second limitation is the absence of a direct assessment of the relationship between brain function and behavioral outcomes. Previous studies have reported that individuals with a history of ELS exhibit poorer performance on response inhibition tasks (Mueller et al., 2010; Wu et al., 2021). However, it remains unclear whether the reduced aperiodic offset in the left pars orbitalis associated with emotional neglect observed in this study directly influences behavioral performance. Further investigation is warranted to determine whether such neurophysiological alterations contribute to deficits in response inhibition.

## 5 Conclusion

The present findings suggest that childhood emotional neglect is associated with a reduction in the aperiodic offset within the left pars orbitalis. This alteration may reflect decreased GABAergic function and/or relatively increased glutamatergic activity, which potentially underlies deficits in inhibitory control and emotion regulation. These findings offer important insights into the long-term impact of early life stress on prefrontal cortical function.

## Data Availability

The raw data supporting the conclusions of this article will be made available by the authors, without undue reservation.

## References

[ref1] Arutiunian V. Arcara G. Buyanova I. Fedorov M. Davydova E. Pereverzeva D. . (2024). Abnormalities in both stimulus-induced and baseline MEG alpha oscillations in the auditory cortex of children with autism Spectrum disorder. Brain Struct. Funct. 229, 1225–1242. doi: 10.1007/s00429-024-02802-7, 38683212

[ref2] Beck A. T. Steer R. A. Ball R. Ranieri W. (1996). Comparison of Beck depression inventories -IA and -II in psychiatric outpatients. J. Pers. Assess. 67, 588–597. doi: 10.1207/s15327752jpa6703_13, 8991972

[ref3] Belyk M. Brown S. Lim J. Kotz S. A. (2017). Convergence of semantics and emotional expression within the IFG pars orbitalis. NeuroImage 156, 240–248. doi: 10.1016/j.neuroimage.2017.04.020, 28400265

[ref4] Bernstein D. Fink L. (1998). Manual for the childhood trauma questionnaire. New York, NY: The Psychological Corporation.

[ref5] Bernstein D. P. Stein J. A. Newcomb M. D. Walker E. Pogge D. Ahluvalia T. . (2003). Development and validation of a brief screening version of the childhood trauma questionnaire. Child Abuse Negl. 27, 169–190. doi: 10.1016/s0145-2134(02)00541-0, 12615092

[ref6] Buzsáki G. Anastassiou C. A. Koch C. (2012). The origin of extracellular fields and currents--EEG, ECoG, LFP and spikes. Nat. Rev. Neurosci. 13, 407–420. doi: 10.1038/nrn3241, 22595786 PMC4907333

[ref7] Carr C. P. Martins C. M. S. Stingel A. M. Lemgruber V. B. Juruena M. F. (2013). The role of early life stress in adult psychiatric disorders: a systematic review according to childhood trauma subtypes. J. Nerv. Ment. Dis. 201, 1007–1020. doi: 10.1097/NMD.0000000000000049, 24284634

[ref8] Chocyk A. Bobula B. Dudys D. Przyborowska A. Majcher-Maślanka I. Hess G. . (2013). Early-life stress affects the structural and functional plasticity of the medial prefrontal cortex in adolescent rats. Eur. J. Neurosci. 38, 2089–2107. doi: 10.1111/ejn.12208, 23581639

[ref9] Davey C. G. Yücel M. Allen N. B. (2008). The emergence of depression in adolescence: development of the prefrontal cortex and the representation of reward. Neurosci. Biobehav. Rev. 32, 1–19. doi: 10.1016/j.neubiorev.2007.04.016, 17570526

[ref10] Dillo W. Göke A. Prox-Vagedes V. Szycik G. R. Roy M. Donnerstag F. . (2010). Neuronal correlates of ADHD in adults with evidence for compensation strategies--a functional MRI study with a go/no-go paradigm. Ger. Med. Sci. 8:Doc09. doi: 10.3205/000098, 20421953 PMC2858877

[ref11] Donoghue T. Haller M. Peterson E. J. Varma P. Sebastian P. Gao R. . (2020). Parameterizing neural power spectra into periodic and aperiodic components. Nat. Neurosci. 23, 1655–1665. doi: 10.1038/s41593-020-00744-x, 33230329 PMC8106550

[ref12] Earl R. J. Ford T. C. Lum J. A. G. Enticott P. G. Hill A. T. (2024). Exploring aperiodic activity in first episode schizophrenia spectrum psychosis: a resting-state EEG analysis. Brain Res. 1840:149052. doi: 10.1016/j.brainres.2024.149052, 38844199

[ref13] Englund J. Haikonen J. Shteinikov V. Amarilla S. P. Atanasova T. Shintyapina A. . (2021). Downregulation of kainate receptors regulating GABAergic transmission in amygdala after early life stress is associated with anxiety-like behavior in rodents. Transl. Psychiatry 11:538. doi: 10.1038/s41398-021-01654-7, 34663781 PMC8523542

[ref14] Fu H. Feng T. Qin J. Wang T. Wu X. Cai Y. . (2018). Reported prevalence of childhood maltreatment among Chinese college students: a systematic review and meta-analysis. PLoS One 13:e0205808. doi: 10.1371/journal.pone.0205808, 30321243 PMC6188789

[ref15] Gaser C. Dahnke R. Thompson P. M. Kurth F. Luders E. The Alzheimer’s Disease Neuroimaging Initiative (2024). CAT: a computational anatomy toolbox for the analysis of structural MRI data. Gigascience 13:giae049. doi: 10.1093/gigascience/giae049 39102518 PMC11299546

[ref16] Gilbert R. Widom C. S. Browne K. Fergusson D. Webb E. Janson S. (2009). Burden and consequences of child maltreatment in high-income countries. Lancet 373, 68–81. doi: 10.1016/s0140-6736(08)61706-7, 19056114

[ref17] Glica A. Wasilewska K. Jurkowska J. Żygierewicz J. Kossowski B. Jednoróg K. (2025). Reevaluating the neural noise in dyslexia using biomarkers from electroencephalography and high-resolution magnetic resonance spectroscopy. eLife 13:eLife.99920. doi: 10.7554/eLife.99920, 40029268 PMC11875536

[ref18] Gondré-Lewis M. C. Warnock K. T. Wang H. June H. L. Jr. Bell K. A. Rabe H. . (2016). Early life stress is a risk factor for excessive alcohol drinking and impulsivity in adults and is mediated via a CRF/GABA (a) mechanism. Stress 19, 235–247. doi: 10.3109/10253890.2016.1160280, 27023221 PMC4962560

[ref19] Gonzalez-Burgos I. Bainier M. Gross S. Schoenenberger P. Ochoa J. A. Valencia M. . (2023). Glutamatergic and GABAergic receptor modulation present unique electrophysiological fingerprints in a concentration-dependent and region-specific manner. eNeuro 10, ENEURO.0406–ENEU22.2023. doi: 10.1523/ENEURO.0406-22.2023, 36931729 PMC10124153

[ref20] Goodwill H. L. Manzano-Nieves G. LaChance P. Teramoto S. Lin S. Lopez C. . (2018). Early life stress drives sex-selective impairment in reversal learning by affecting Parvalbumin interneurons in orbitofrontal cortex of mice. Cell Rep. 25, 2299–2307.e4. doi: 10.1016/j.celrep.2018.11.010, 30485800 PMC6310486

[ref21] Hill A. T. Clark G. M. Bigelow F. J. Lum J. A. G. Enticott P. G. (2022). Periodic and aperiodic neural activity displays age-dependent changes across early-to-middle childhood. Dev. Cogn. Neurosci. 54:101076. doi: 10.1016/j.dcn.2022.101076, 35085871 PMC8800045

[ref22] Holland F. H. Ganguly P. Potter D. N. Chartoff E. H. Brenhouse H. C. (2014). Early life stress disrupts social behavior and prefrontal cortex parvalbumin interneurons at an earlier time-point in females than in males. Neurosci. Lett. 566, 131–136. doi: 10.1016/j.neulet.2014.02.023, 24565933 PMC4476267

[ref23] Inoue Y. Sakihara K. Gunji A. Ozawa H. Kimiya S. Shinoda H. . (2012). Reduced prefrontal hemodynamic response in children with ADHD during the go/NoGo task: a NIRS study: a NIRS study. Neuroreport 23, 55–60. doi: 10.1097/WNR.0b013e32834e664c 22146580

[ref24] Irie K. Ohta K.-I. Ujihara H. Araki C. Honda K. Suzuki S. . (2023). An enriched environment ameliorates the reduction of parvalbumin-positive interneurons in the medial prefrontal cortex caused by maternal separation early in life. Front. Neurosci. 17:1308368. doi: 10.3389/fnins.2023.1308368, 38292903 PMC10825025

[ref25] Jin X. Xu B. Xu R. Yin X. Yan S. Zhang Y. . (2023). The influence of childhood emotional neglect experience on brain dynamic functional connectivity in young adults. Eur. J. Psychotraumatol. 14:2258723. doi: 10.1080/20008066.2023.2258723, 37736668 PMC10519269

[ref26] Kaiser S. Unger J. Kiefer M. Markela J. Mundt C. Weisbrod M. (2003). Executive control deficit in depression: event-related potentials in a go/Nogo task. Psychiatry Res. 122, 169–184. doi: 10.1016/s0925-4927(03)00004-0, 12694891

[ref27] Knudsen E. I. (2004). Sensitive periods in the development of the brain and behavior. J. Cogn. Neurosci. 16, 1412–1425. doi: 10.1162/0898929042304796, 15509387

[ref28] Kojima M. Furukawa T. A. Takahashi H. Kawai M. Nagaya T. Tokudome S. (2002). Cross-cultural validation of the Beck depression inventory-II in Japan. Psychiatry Res. 110, 291–299. doi: 10.1016/s0165-1781(02)00106-3, 12127479

[ref29] Kolb B. Harker A. Gibb R. (2017). Principles of plasticity in the developing brain. Dev. Med. Child Neurol. 59, 1218–1223. doi: 10.1111/dmcn.13546, 28901550

[ref30] Malave L. van Dijk M. T. Anacker C. (2022). Early life adversity shapes neural circuit function during sensitive postnatal developmental periods. Transl. Psychiatry 12:306. doi: 10.1038/s41398-022-02092-9, 35915071 PMC9343623

[ref31] Manning J. R. Jacobs J. Fried I. Kahana M. J. (2009). Broadband shifts in local field potential power spectra are correlated with single-neuron spiking in humans. J. Neurosci. 29, 13613–13620. doi: 10.1523/JNEUROSCI.2041-09.2009, 19864573 PMC3001247

[ref32] McLaughlin K. A. Fox N. A. Zeanah C. H. Nelson C. A. (2011). Adverse rearing environments and neural development in children: the development of frontal electroencephalogram asymmetry. Biol. Psychiatry 70, 1008–1015. doi: 10.1016/j.biopsych.2011.08.006, 21962332 PMC3207006

[ref33] McSweeney M. Morales S. Valadez E. A. Buzzell G. A. Yoder L. Fifer W. P. . (2023). Age-related trends in aperiodic EEG activity and alpha oscillations during early- to middle-childhood. NeuroImage 269:119925. doi: 10.1016/j.neuroimage.2023.119925, 36739102 PMC10701700

[ref34] Miller K. J. Hermes D. Honey C. J. Hebb A. O. Ramsey N. F. Knight R. T. . (2012). Human motor cortical activity is selectively phase-entrained on underlying rhythms. PLoS Comput. Biol. 8:e1002655. doi: 10.1371/journal.pcbi.1002655, 22969416 PMC3435268

[ref35] Miskovic V. Schmidt L. A. Georgiades K. Boyle M. MacMillan H. L. (2009). Stability of resting frontal electroencephalogram (EEG) asymmetry and cardiac vagal tone in adolescent females exposed to child maltreatment. Dev. Psychobiol. 51, 474–487. doi: 10.1002/dev.20387, 19629997

[ref36] Mueller S. C. Maheu F. S. Dozier M. Peloso E. Mandell D. Leibenluft E. . (2010). Early-life stress is associated with impairment in cognitive control in adolescence: an fMRI study. Neuropsychologia 48, 3037–3044. doi: 10.1016/j.neuropsychologia.2010.06.013, 20561537 PMC2916226

[ref37] Murthy S. Kane G. A. Katchur N. J. Lara Mejia P. S. Obiofuma G. Buschman T. J. . (2019). Perineuronal nets, inhibitory interneurons, and anxiety-related ventral hippocampal neuronal oscillations are altered by early life adversity. Biol. Psychiatry 85, 1011–1020. doi: 10.1016/j.biopsych.2019.02.021, 31027646 PMC6590696

[ref38] Nakajima M. Hori H. Itoh M. Lin M. Kawanishi H. Narita M. . (2022). Validation of childhood trauma questionnaire-short form in Japanese clinical and nonclinical adults. Psychiatry Res. Commun. 2:100065. doi: 10.1016/j.psycom.2022.100065

[ref39] Nakazato K. Shimonaka Y. (1989). The Japanese state-trait anxiety inventory: age and sex differences. Percept. Mot. Skills 69, 611–617. doi: 10.2466/pms.1989.69.2.611, 2813009

[ref40] Nanni V. Uher R. Danese A. (2012). Childhood maltreatment predicts unfavorable course of illness and treatment outcome in depression: a meta-analysis. Am. J. Psychiatry 169, 141–151. doi: 10.1176/appi.ajp.2011.11020335, 22420036

[ref41] Nelson J. Klumparendt A. Doebler P. Ehring T. (2017). Childhood maltreatment and characteristics of adult depression: meta-analysis. Br. J. Psychiatry 210, 96–104. doi: 10.1192/bjp.bp.115.180752, 27908895

[ref42] Nemeroff C. B. (2016). Paradise lost: the neurobiological and clinical consequences of child abuse and neglect. Neuron 89, 892–909. doi: 10.1016/j.neuron.2016.01.019, 26938439

[ref43] Pechtel P. Pizzagalli D. A. (2011). Effects of early life stress on cognitive and affective function: an integrated review of human literature. Psychopharmacology 214, 55–70. doi: 10.1007/s00213-010-2009-2, 20865251 PMC3050094

[ref44] Salokangas R. K. R. (2022). Emotional neglect in childhood is common and associates with adult mental ill health. Nord. J. Psychiatry 75:Suppl. 1. doi: 10.1080/08039488.2021.2019939

[ref45] Salvatore S. V. Lambert P. M. Benz A. Rensing N. R. Wong M. Zorumski C. F. . (2024). Periodic and aperiodic changes to cortical EEG in response to pharmacological manipulation. J. Neurophysiol. 131, 529–540. doi: 10.1152/jn.00445.2023, 38323322 PMC11305649

[ref46] Shaw G. A. Dupree J. L. Neigh G. N. (2020). Adolescent maturation of the prefrontal cortex: role of stress and sex in shaping adult risk for compromise. Genes Brain Behav. 19:e12626. doi: 10.1111/gbb.12626, 31769158

[ref47] Sheikhattari P. Stephenson R. Assasi N. Eftekhar H. Zamani Q. Maleki B. . (2006). Child maltreatment among school children in the Kurdistan Province, Iran. Child Abuse Negl. 30, 231–245. doi: 10.1016/j.chiabu.2005.10.009, 16524629

[ref48] Spielberger C. D. Gonzalez-Reigosa F. Martinez-Urrutia A. Natalicio L. F. S. Natalicio D. S. (1971). The state-trait anxiety inventory. Int. J. Psychol. 5, 145–158. doi: 10.30849/rip/ijp.v5i3

[ref49] Swick D. Ashley V. Turken A. U. (2008). Left inferior frontal gyrus is critical for response inhibition. BMC Neurosci. 9:102. doi: 10.1186/1471-2202-9-102, 18939997 PMC2588614

[ref50] Tadel F. Baillet S. Mosher J. C. Pantazis D. Leahy R. M. (2011). Brainstorm: a user-friendly application for MEG/EEG analysis. Comput. Intell. Neurosci. 2011:879716. doi: 10.1155/2011/879716, 21584256 PMC3090754

[ref51] Teicher M. H. Samson J. A. (2016). Annual research review: enduring neurobiological effects of childhood abuse and neglect. J. Child Psychol. Psychiatry 57, 241–266. doi: 10.1111/jcpp.12507, 26831814 PMC4760853

[ref52] Vanderwert R. E. Marshall P. J. Nelson C. A. 3rd Zeanah C. H. Fox N. A. (2010). Timing of intervention affects brain electrical activity in children exposed to severe psychosocial neglect. PLoS One 5:e11415. doi: 10.1371/journal.pone.0011415, 20617175 PMC2895657

[ref53] Wang Y.-P. Gorenstein C. (2013). Psychometric properties of the Beck depression inventory-II: a comprehensive review. Rev. Bras. Psiquiatr. 35, 416–431. doi: 10.1590/1516-4446-2012-1048, 24402217

[ref54] Weisbrod M. Kiefer M. Marzinzik F. Spitzer M. (2000). Executive control is disturbed in schizophrenia: evidence from event-related potentials in a go/NoGo task. Biol. Psychiatry 47, 51–60. doi: 10.1016/s0006-3223(99)00218-8, 10650449

[ref55] Winawer J. Kay K. N. Foster B. L. Rauschecker A. M. Parvizi J. Wandell B. A. (2013). Asynchronous broadband signals are the principal source of the BOLD response in human visual cortex. Curr. Biol. 23, 1145–1153. doi: 10.1016/j.cub.2013.05.001, 23770184 PMC3710543

[ref56] Woronko S. E. Li M. Scott J. N. Jr. Kuhn M. Esfand S. M. Bogdanov M. . (2025). Alterations in aperiodic neural activity associated with major depressive disorder. Nat. Ment. Health 3, 1181–1190. doi: 10.1038/s44220-025-00494-4 PMC13455338 42577840

[ref57] Wu J. Liu Y. Fang H. Qin S. Kohn N. Duan H. (2021). The relationship between childhood stress and distinct stages of dynamic behavior monitoring in adults: neural and behavioral correlates. Soc. Cogn. Affect. Neurosci. 16, 937–949. doi: 10.1093/scan/nsab041, 33830244 PMC8421694

[ref58] Xu B. Wei S. Yin X. Jin X. Yan S. Jia L. (2023). The relationship between childhood emotional neglect experience and depressive symptoms and prefrontal resting functional connections in college students: the mediating role of reappraisal strategy. Front. Behav. Neurosci. 17:927389. doi: 10.3389/fnbeh.2023.927389, 36969801 PMC10037214

